# Targeting Adenosine Signaling in Parkinson's Disease: From Pharmacological to Non-pharmacological Approaches

**DOI:** 10.3389/fnins.2017.00658

**Published:** 2017-11-23

**Authors:** Luiza R. Nazario, Rosane S. da Silva, Carla D. Bonan

**Affiliations:** Laboratório de Neuroquímica e Psicofarmacologia, Departamento de Biologia Celular e Molecular, Faculdade de Biociências, Pontifícia Universidade Católica do Rio Grande do Sul, Porto Alegre, Brazil

**Keywords:** adenosine, A_2A_AR, dopaminergic system, neurodegeneration, Parkinson disease

## Abstract

Parkinson's disease (PD) is one of the most prevalent neurodegenerative disease displaying negative impacts on both the health and social ability of patients and considerable economical costs. The classical anti-parkinsonian drugs based in dopaminergic replacement are the standard treatment, but several motor side effects emerge during long-term use. This mini-review presents the rationale to several efforts from pre-clinical and clinical studies using adenosine receptor antagonists as a non-dopaminergic therapy. As several studies have indicated that the monotherapy with adenosine receptor antagonists reaches limited efficacy, the usage as a co-adjuvant appeared to be a promising strategy. The formulation of multi-targeted drugs, using adenosine receptor antagonists and other neurotransmitter systems than the dopaminergic one as targets, have been receiving attention since Parkinson's disease presents a complex biological impact. While pharmacological approaches to cure or ameliorate the conditions of PD are the leading strategy in this area, emerging positive aspects have arisen from non-pharmacological approaches and adenosine function inhibition appears to improve both strategies.

## General aspects of parkinson's disease

Parkinson's disease (PD) is the second most prevalent chronic neurodegenerative disease, affecting more than 1% of the elderly population, with diagnostic confirmation occurring when the loss of dopaminergic neurons in the striatum is close to 80% (de Rijk et al., 2000). PD is also diagnosed in people less than 40 years old, named early-onset PD (Crosiers et al., 2011). PD is associated with the formation of Lewy bodies and neurites (Braak et al., 2003), mainly composed of aggregated forms of α-synuclein (Spillantini et al., 1998). The loss of dopaminergic neurons causes a reduction in the release of dopamine, leading to motor symptoms such as bradykinesia, rigidity, imbalance and tremor (Jankovic, 2008). PD presents in sporadic and familial forms. The risk factors involved in the development of PD are both genetic and environmental (Mortimer et al., 2012; Noyce et al., 2012; Van der Mark et al., 2012; Pezzoli and Cereda, 2013). The familial form, with specific genetic targets, represents less than 10% of PD cases (Dawson and Dawson, 2010). The genetic aspects of the disease are linked to mutations in several genes related to a multitude of cellular mechanisms, such as protein aggregation, protein and membrane trafficking, lysosomal autophagy, immune response, synaptic function, endocytosis, inflammation, and metabolic pathways (Redenšek et al., 2017). The genes *SNCA* (PARK1), *UCHL1* (PARK5), *LRRK2* (PARK8), GIGYF2 (PARK11), *OMI/HTRA2* (PARK13), VPS35 (PARK17), and EIF4G1 (PARK18) result in autosomal dominant PD, and PRKN (PARK2), DJ-1 (PARK7), ATP13A2 (PARK9), PLA2G6 (PARK14), FBX07 (PARK15), DNJC6 (PARK19), and SYNJ1 (PARK20) causes autosomal recessive PD (Lautier et al., 2008; Di Fonzo et al., 2009; Klein and Westenberger, 2012; Deng et al., 2015; Bartonikova et al., 2016; Miki et al., 2017; Scott et al., 2017). The gene contribution from other loci (PARK 3, 10, 12, and 16) is under investigation (Dawson and Dawson, 2010). However, a putative causative mutation in the gene that encodes the A_1_ adenosine receptor, located in the locus PARK16, has been related to susceptibility to PD (Jaberi et al., 2016). Among the environmental contributors to PD development are occupational exposure of pesticides, such as Rotenone and Paraquat, infection by *Helicobacter* and HCV, low body weight and sedentary lifestyle (McCarthy et al., 2004; Villar-Cheda et al., 2009; Golabi et al., 2017; Sharma and Lewis, 2017; Shen et al., 2017).

## The relationship of adenosine and dopamine signaling

Adenosine affects dopaminergic signaling through receptor heteromer formations and shared intracellular pathways. Adenosine is a neuromodulator that acts through the A_1_ (A_1_AR) and A_3_ (A_3_AR) inhibitory adenosine receptors and A_2A_ (A_2A_AR) and A_2B_ (A_2B_AR) excitatory adenosine receptors (Ralevic and Burnstock, 1998). D1 (D_1_DR) and D2 (D_2_DR) dopamine receptors are found co-localized with A_2A_AR and A_1_AR, mGluR_5_ and NMDA (Hillion et al., 2002; Lee et al., 2002; Beggiato et al., 2016). The dopamine-adenosine receptor heteromers are constituted mainly of D_1_DR/A_1_AR and D_2_DR/A_2A_AR, displaying antagonistic properties. A_1_AR agonist decreases the binding potential of dopamine to D_1_DR, and reduces the D_1_DR-induced cAMP production, while A_1_AR antagonists activate D_1_DR increasing cAMP levels (Ferré et al., 1998). A_3_AR activation appears to have some influence on dopamine release and vesicular transport, while no functional impacts have been registered in dopamine receptors (Gołembiowska and Zylewska, 1998; Björklund et al., 2008; Shen et al., 2011).

The heteromerization of D_2_DR/A_2A_AR is one of the most studied receptors interaction. A_2A_AR agonists reduce the *in vitro* affinity of the D_2_DR agonist through an increase in D_2_DR Kd without affecting receptor density (Ferré et al., 1991). *In vivo* studies confirmed these findings since the administration of A_2A_AR antagonist increased the effects of the D_2_DR agonist in the rat striatum and basal ganglia, while the action of A_2A_AR agonists was opposite (Hillefors-Berglund et al., 1995; Strömberg et al., 2000). This heteromerization was confirmed through co-immunoprecipitation, fluorescence resonance energy, bioluminescence resonance energy transfer and *ex vivo* proximity ligation studies (Hillion et al., 2002; Canals et al., 2003; Trifilieff et al., 2011; Fernández-Dueñas et al., 2015). Studies with PET in the human brain showed the increased binding of a D_2_DR antagonist, after the administration of caffeine, a nonselective antagonist of adenosine receptors (Volkow et al., 2015).

The interaction between adenosinergic and dopaminergic receptors has been described as intramembrane, involving direct interaction between receptors, or the modulation of G-proteins and the consequent influence on cAMP-dependent proteins (Fuxe et al., 1998; Ferré et al., 2001; Hillion et al., 2002; Fredholm and Svenningsson, 2003). The administration of D_2_DR antagonists can reduce the cAMP production by A_2A_AR and the D_2_ agonist administration induces increase in cAMP levels by A_2A_AR (Vortherms and Watts, 2004; Botsakis et al., 2010). A_2A_AR stimulation, *in vitro*, causes the phosphorylation and activation of DARPP-32, which can be inhibited by D_2_DR activation (Nishi et al., 1997). A_2A_AR antagonists increase D_2_DR-dependent regulation of *c-fos*, which is more intense when dopaminergic neurodegeneration is presented (Pollack and Fink, 1995; Svenningsson et al., 1999). Compelling evidence for the impairment of D_2_DR/A_2A_AR oligomers in the striatum of rats was obtained in experimental Parkinsonism induced by 6-hydroxydopamine (6-OHDA) (Fernández-Dueñas et al., 2015). The ventral striopallidal GABA pathway appears to be a target of mGlu_5_R/D_2_DR/A_2A_AR interactions. The co-administration of A_2A_AR and mGlu_5_R agonist enhances GABA release compared with mGlu_5_R agonist alone, and this effect decreases with the administration of D_2_DR agonists (Díaz-Cabiale et al., 2002). In addition, D_2_DR/A_2A_AR controls NMDA-mediated excitation in neurons from the nucleus accumbens through a direct protein–protein interaction (Azdad et al., 2009).

## Support for the A_2A_AR antagonism hypothesis from animal studies

The co-expression of D_2_DR/A_2A_AR receptors and their close functional and structural association in the striatopallidal GABAergic neurons reveals sites for therapeutic intervention and has received attention in the last three decades (Fink et al., 1992; Kase, 2001; Kelsey et al., 2009). The non-specific blockade of adenosine receptors by methylxanthines produces contralateral rotations in animals with dopaminergic lesions induced by 6-OHDA, since contralateral rotations have been related to an indirect stimulation of dopamine receptors in the lesioned area (Watanabe et al., 1981; Herrera-Marschitz et al., 1988).

During the late 1990s and early 2000s, exciting results from animal models of Parkinsonism indicated that A_2A_AR antagonism improves motor activity by reducing the postsynaptic effects of dopamine depletion. Caffeine neuroprotection against 1-methyl-4-phenyl-1,2,3,6-tetrahydropyridine (MPTP)-induced lesion showed to be especially dependent on A_2A_AR from the striatal neurons, but not exclusively (Chen et al., 2001; Xu et al., 2016). The A_2A_AR antagonist KW6002 (Istradefylline) was shown to be powerful enough to increase locomotion activity and potentiate dopaminergic agonist motor effects in MPTP- and 6-OHDA-lesioned animals (Kanda et al., 1998, 2000; Grondin et al., 1999; Koga et al., 2000; Bibbiani et al., 2003). The anti-parkinsonian effects of KW6002 and similar drugs, such as KW17837, appear to be dose-dependent, effective in the postsynapse and beyond the direct effect on the dopaminergic system, and act on glutamatergic/gabaergic neurotransmission and monoamine oxidase activity (Bibbiani et al., 2003; Petzer et al., 2003; Tanganelli et al., 2004; Orru et al., 2011). MSX-3, a water-soluble precursor of the highly specific A_2A_AR antagonist MSX-2, which exhibits greater potency for A_2A_AR than KW6002, appeared to be a candidate of monotherapy since it alleviates the symptomatic parkinsonian locomotor deficiency in a genetic model of dopaminergic degeneration (Yang et al., 2007; Marcellino et al., 2010).

While some studies advocated that A_2A_AR antagonism, as a monotherapy, could reach a mildly lower or similar efficacy of L-DOPA treatment without inducing dyskinesia (Grondin et al., 1999; Pinna et al., 2007), the promisor effect of these drugs appeared to be when co-administrated with L-DOPA, simultaneously inhibiting A_2A_AR and activating D_2_DR. A_2A_AR-knockout animals demonstrated weak and transitory rotational sensitization and no sensitized grooming as a response to L-DOPA (Fredduzzi et al., 2002). The blockade of adenosine receptors by caffeine promoted additive or synergistic interactions with L-DOPA (Yu et al., 2006), whereas the co-administration of specific A_2A_AR antagonists, such as KW6002, ST1535, and L-DOPA, potentiated the anti-parkinsonian effect of L-DOPA without exacerbating dyskinesia (Kanda et al., 2000; Koga et al., 2000; Bibbiani et al., 2003; Matsuya et al., 2007; Tronci et al., 2007). However, some studies using several A_2A_AR antagonists, such as SCH4123-48, BIIB014 (Vipanedant), KW6002 and caffeine, when administered concomitantly and chronically with L-DOPA, failed to avoid dyskinesia (Jones et al., 2013).

**Figure 1:**
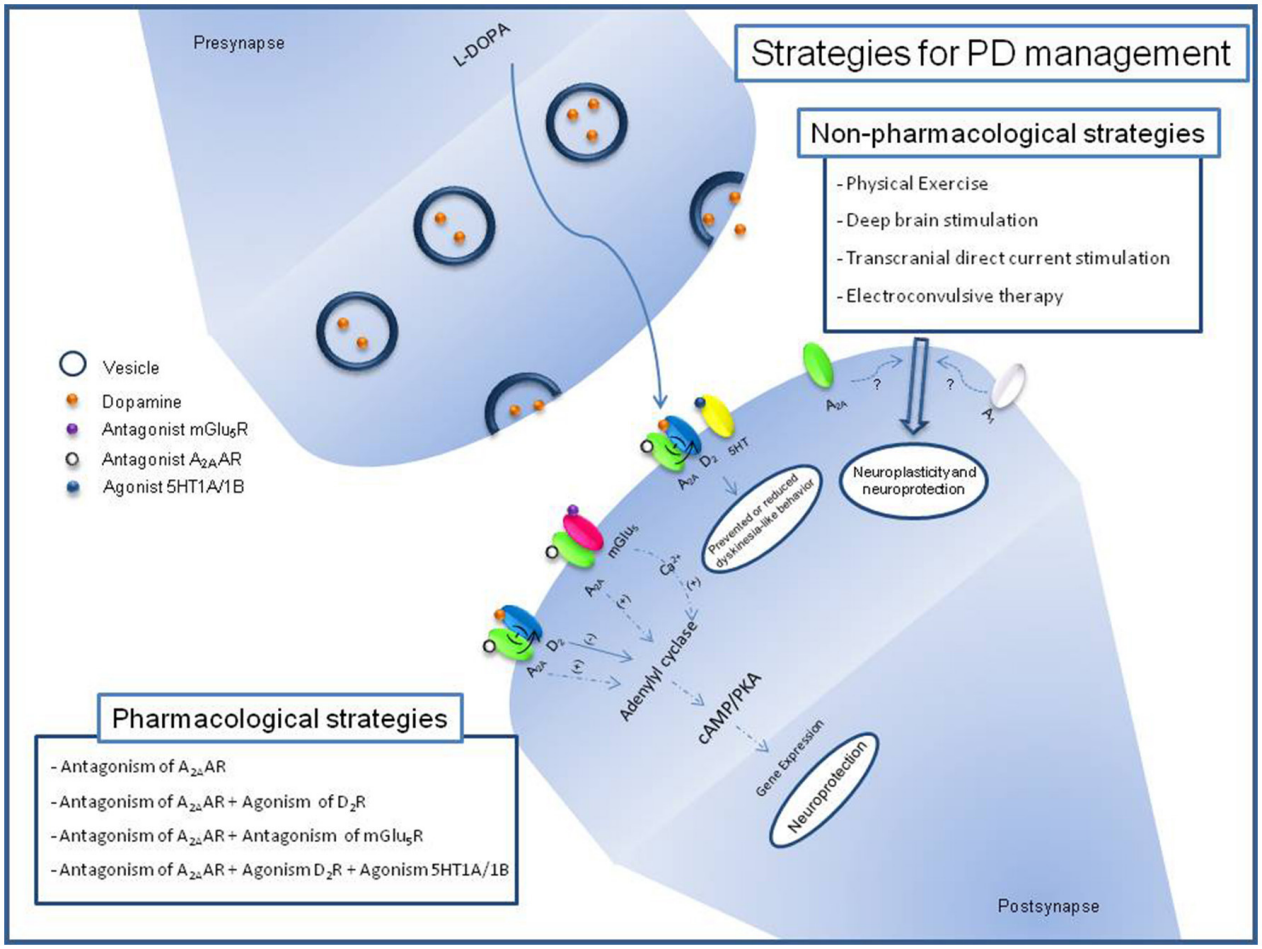
Schematic description of pharmacological and non-pharmacological strategies for PD management and its relation with adenosinergic signaling. Block of A_2A_AR by antagonist induces reduction of positive effects over Adenylyl cyclase and negative effects over D2R signaling. Block of mGlu_5_R reduces its positive effects over Adenilyl cyclase through release of Ca^2+^. Recent studies with non-phamacological strategies for PD have been related it with adenosine receptors expression.

The mechanism behind the effects of A_2A_AR antagonists alone or as co-adjuvant drugs appears to beyond actions on dopaminergic system (Fuxe et al., 2009; Maggio et al., 2009; Figure 1). The A_2A_AR exerts its neuronal activity in the striatum in a manner that is partially independent of D_2_Rs (Chen et al., 2001). Actually, KW6002 decreases the neuronal activity of the striatopallidal indirect pathway in the absence of D_2_R-mediated signaling (Aoyama et al., 2000). Dopaminergic neurodegeneration induced by transgenic mutant human α-synuclein is prevented in mice lacking the A_2A_AR reinforcing the potential of shared downstream pathways (Ferraro et al., 2012). However, the adenylate cyclase activity did not differ in a genetic model of PD, suggesting that coupling to G-proteins of dopaminergic and adenosinergic receptors should be a target (Botsakis et al., 2010). Regional differences appear in the anti-parkinsonian ability of A_2A_AR antagonism, since caffeine given at or before MPTP exposure blocks the nigral neurodegenerative process without restoring the striatal nerve terminal neurochemical features (Sonsalla et al., 2012). Motor sensitization developed in unilaterally 6-OHDA-lesioned rats submitted to L-DOPA has been associated with an overexpression of the GABA-synthesizing enzyme glutamic acid decarboxylase, dynorphin, and enkephalin mRNAs in the striatal efferent indirect pathway (Fink et al., 1992; Tronci et al., 2007). The impact of A_2A_AR antagonism over enkephalin content seems to promote motor recovery in D_2_DR-knockout animals, but did not promote changes in the preproenkephalin mRNA in a 6-OHDA model (Fink et al., 1992; Aoyama et al., 2000). The functional relation of D_2_DR/A_2A_AR in striatal medium spiny neurons appears to receive contributions of cholinergic signaling with consequences for the anti-tremor benefits of A_2A_AR antagonists (Simola et al., 2006; Tozzi et al., 2011; Salamone et al., 2013). The existence of A_2A_AR/mGlu_5_R heteromers and shared intracellular cascades steps, such as the stimulation of DARPP32 phosphorylation, increase in cAMP levels and elevated *c-fos* expression, provides clues to the possible contribution of glutamatergic and adenosinergic signaling to the beneficial effects of adenosine receptor antagonism (Nash and Brotchie, 2000; Kachroo et al., 2005). Effects resembling akinesia in 6-OHDA-lesioned rats were fully reversed by either a single treatment of an A_2A_AR antagonist or an mGlu_5_R antagonist at higher doses, or by a combined treatment with ineffective doses of each compound (Coccurello et al., 2004). Increased A_2A_AR mRNA levels, decreased DARPP-32 phosphorylation and increased phosphorylation of ERK1/2 appeared in 6-OHDA-lesioned rats that display L-DOPA motor sensitization (Tomiyama et al., 2004; Song et al., 2009). This altered downstream signaling pathway is recovered by CSC (8-(3-chlorostryryl) caffeine), an A_2A_AR antagonist (Song et al., 2009). Amelioration of motor response by A_2A_AR antagonism seems to be accompanied by the rescue of dopamine, dopamine metabolites, glutamate, and GABA striatal levels as well as the reversal of astroglial and microglial activation and antioxidant properties with beneficial outcomes on cognition (Aguiar et al., 2008; Gołembiowska et al., 2013; Uchida et al., 2014).

Prodrugs such as DP-L-A2AANT were designed to conjugate the beneficial effects against dopaminergic degeneration obtained by the combined action of dopamine and A_2A_AR antagonists in central nervous system (Dalpiaz et al., 2012). In addition to the potential dual action on adenosinergic and dopaminergic systems, the complimentary action on glutamatergic and adenosinergic systems appeared as prospective targets for dual anti-parkinsonian approaches. The combination of A_2A_AR antagonists and NR2B or mGlu_5_R antagonists has demonstrated attractive effects on motor activity with potential in the treatment of PD (Michel et al., 2014, 2015; Beggiato et al., 2016). A_2A_AR–CB_1_-D_2_DR-receptor-heteromer has been suggested as a component of motor alterations associated with dyskinesia and a possible target of multi-targeted drugs (Bonaventura et al., 2014; Pinna et al., 2014). The effects of caffeine-derived compounds over A_2A_AR and that of monoamine oxidase B have revealed that these proteins are targets for synergistic action with benefits on dopaminergic degeneration (Petzer and Petzer, 2015). Sulphanylphthalimides are also presented as a dual-targeted-direct compound acting in A_1_AR and monoamine oxidase B (Van der Walt et al., 2015). The association of L-dopa, serotonin 5-HT1A/1B receptor agonist and A_2A_AR antagonist also demonstrated a promissory strategy in 6-OHDA-lesioned rats exhibiting prevented or reduced dyskinetic-like behavior without impairing motor activity (Pinna et al., 2016).

## Support for the A_2A_AR antagonism hypothesis from clinical tests

The A_2A_AR biding sites and mRNA levels in PD patients with dyskinesia are increased in striatopallidal pathway neurons in relation to healthy patients (Martinez-Mir et al., 1991; Calon et al., 2004). These data, in association with the experimental benefits of A_2A_AR antagonists in dopaminergic degenerative diseases increased the enthusiasm regarding non-dopaminergic drug development. Table 1 updates the clinical trials assigned in the EUA and European Union using adenosine receptor antagonists. Istradefylline had long-term tolerability and safety, including as an adjuvant therapy to levodopa (Hauser et al., 2003; Stacy et al., 2008). In 2008, US Food and Drug Administration issued a non-approvable letter to the use of Istradefylline in humans based in the concern if the efficacy findings support clinical utility of Istradefylline in patients with PD. However, Kyowa Hakko Kirin has received approval for the use of Istradefylline as adjunctive therapy in Japan (Dungo and Deeks, 2013; Mizuno et al., 2013). After the additional data request, a 12-week randomized study to evaluate oral Istradefylline in subjects with moderate to severe PD ended with disappointing results, since Istradefylline did not change the off time per day (NCT01968031). However, a clinical trial is currently open (NCT02610231). Preladenant was evaluated as monotherapy to patients with early PD since it reduced the mean daily off time in a phase II study; however, no evidence has supported its efficacy in phase III studies (Hauser, 2011; Stocchi et al., 2017). BIIB014 and SCH900800 also failed to prove efficacy in clinical trials, while Tozadenant showed a mean daily off time reduction accompanied by adverse events of dyskinesia, nausea, and dizziness (Hauser et al., 2014). A safety and efficacy study of Tozadenant to treat end of dose wearing off in PD patients using L-DOPA is currently open (NCT02453386). Multiple epidemiological studies indicate that caffeine is able to prevent PD development (Ross et al., 2000; Ascherio et al., 2001). In a pilot study of caffeine for daytime sleepiness in PD, there was evident benefit on the motor manifestations of disease with no adverse effects (Postuma et al., 2012). Recently, a clinical trial has aimed to evaluate the efficacy of caffeine for motor and non-motor aspects of disease (NCT01738178). Nowadays, changing the dose and frequency of daily drug taking had no benefits in the use of adenosine receptor antagonists as a monotherapy or as an adjuvant of current Parkinsonism treatment.

**Table 1:** A_2A_AR antagonists under clinical investigation for Parkinson's disease.

**Drug**	**Sponsor**	**Identifier number (year)**	**Parkinson's disease patient condition**	**Outcome measures (dose tested)**	**Phase**	**Status**	**Results**
**Istradefylline (KW6002)**	Kyowa Hakko Kirin Co., Ltd	NCT02610231^*^ (2015)	Moderate to severe disease	Safety and tolerability (20 or 40 mg oral daily)	III	Active – not recruiting	–
		NCT01968031^*^ (2013) 2013-002254-70^**^ (2014)	Moderate to severe disease	Efficacy and safety (20 or 40 mg daily)	III	Completed	No change in the OFF time
		NCT00957203^*^ (2009)	Advanced disease treated with levodopa	Long-term safety and efficacy (20 or 40 mg daily)	III	Completed	
		NCT00955526^*^ (2009)	Levodopa-treated	Efficacy in reducing the mean total hours of awake time per day spent in the OFF state (20 or 40 mg daily)	III	Completed	Reduction in daily OFF time
		NCT00456794^*^ (2007)	Advanced disease treated with levodopa/carbidopa	Safety and efficacy compared with placebo in subjects with OFF-time (20 and 60 mg daily)	II	Completed	Significant reduction in OFF time, and was well tolerated as adjunctive treatment to levodopa
		NCT00456586^*^ (2007)	Advanced disease treated with levodopa/carbidopa	Safety and efficacy compared with placebo in subjects with OFF phenomena (40 mg daily)	II	Completed	Istradefylline was safe, well toler-ated, and effective at improving end-of-dose wearing
		NCT00455507^*^ (2007)	Advanced disease treated with levodopa	Efficacy for reducing the mean total hours of awake time per day spent in the OFF state(20 or 40 mg daily)	II	Completed	
		2004-002844-93^**^ (2005)	Motor response complications on levodopa therapy	Long-term tolerability and safety (20 or 40 mg daily)	III	Completed	Istradefylline was well tolerated as adjunctive therapy to levodopa for subjects with Parkinson's disease
		NCT00250393^*^ (2005)	Not specified	Change in Unified Parkinson's Disease Rating Scale (UPDRS) part-III (Motor examination) (40 mg daily)	II	Completed	
		NCT00203957^*^ (2005)	Motor response complications on levodopa	Confirmation of long term tolerability and safety (20 or 40 mg daily)	III	Completed	
		NCT00199420^*^ (2005)	Aadvanced disease treated with levodopa	Percentage of OFF time (10, 20 or 40 mg daily)	III	Completed	
		NCT00199407^*^ (2005)	Advanced disease treated with levodopa	Efficacy for reducing the percentage of OFF time (20 mg daily)	III	Completed	
		NCT00199394^*^ (2005)	Advanced disease treated with levodopa	Percentage of awake time spent in the OFF state (40 mg daily)	III	Completed	
		NCT00199381^*^ (2005)	Patients who have recently completed one year of treatment with istradefylline	Long-term tolerability and safety (20 or 40 mg daily)	III	Completed	The sponsor decided to terminate the study early (not for safety reasons)
		NCT00199368^*^ (2005)	Patients with motor response complications on levodopa therapy. Who have completed prior istradefylline studies	Safety Study (20 or 40 mg daily)	III	Completed	
		NCT00199355^*^ (2005)	Advanced disease treated with levodopa /DCI.	OFF time (20 or 40 mg daily)	II		
	NINDS	NCT00006337^*^ (2000)	Not specified	Effects on symptoms and dyskinesias	II	Completed	
**SCH900800**	Merck Sharp & Dohme Corp.	NCT01500707^*^ (2011)	Moderate to severe disease treated with levodopa	Pharmacokinetics of SCH 900800 (20 mg daily)	I	Study withdrawn	-
**Preladenant (SCH 420814)**	Merck Sharp & Dohme Corp.	NCT01294800^*^ (2011)	Moderate to severe disease experiencing motor fluctuations and receiving levodopa	Efficacy on “off” time (2, 5, 10 mg twice/day)	II	Completed	Change from baseline in mean “Off” time
		NCT01227265^*^ (2010)	Moderate to severe disease	Efficacy and safety (2-5 mg twice/day)	III	Completed	Not superior to placebo in reducing off time from baseline
		NCT01155479^*^ (2010)	Early Parkinson's disease	Efficacy and safety (2,5, 10 mg twice/day)	III	Completed	Change from baseline in motor impairments and disability
		2009-015161-31^**^ (2010)	Moderate to severe disease	Efficacy and safety (2,5, 10 mg twice/day)	III	Completed	
		2009-015162-57^**^ (2010)	Moderate to severe disease	Extension study (2,5, 10 mg twice/day)	III	Study withdrawn	Lack of efficacy in the parent studies.
		NCT01155466^*^ (2010)	Moderate to severe disease	Stability in levodopa dose (2, 5, 10 mg twice/day)	III	Completed	No change from baseline in mean “Off” Time
		2009-013552-72^**^ (2010)	Early Parkinson's disease	Dose-range-finding efficacy and safety (2, 5, or 10 mg twice/day)	III	Completed	No statistically significant or clinically meaningful difference vs. placebo
		NCT01215227^*^ (2010)	Moderate to severe disease	Long-term safety and tolerability from patients of NCT01155466 and NCT01227265 (2, 5, 10 mg twice/day)			Terminated early due to the lack of efficacy in the parent studies NCT1155466 and NCT01227265
		NCT00845000^*^ (2009)	Levodopa treated	Effects on the dyskinesia and antiparkinsonian actions of a levodopa infusion (10 or 100 mg daily)	I	Completed	
		NCT00537017^*^ (2007)	Moderate to severe disease	Long term safety (5 mg twice daily)	II	Completed	Long-term preladenant treatment (5 mgtwice a day) was well tolerated and provided sustained OFF time reductions and ON time increases
		NCT00406029^*^ (2006)	Not specified	Efficacy and safety when used together with a stable dose of L-dopa/dopa decarboxylase (1, 2, 5, and 10 mg twice a day)	II	Completed	Mean daily off time reduced (5 and 10 mg)
**Tozadenant (SYN115)**	Biotie Therapies Inc.	NCT03051607^*^ 2016-003961-25^**^ (2017)	Experiencing end of dose “Wearing-Off”	Safety and tolerability(120 mg oral twice daily)	III	Recruiting	-
		2014-005630-60 ^**^ (2015)	Levodopa-treated experiencing end-of-dose “Wearing-Off”	Efficacy and safety as adjunctive therapy to levodopa (60 mg oral daily)	III	Active	-
		2011-005054-59 ^**^ (2013)	Experiencing end of dose ”Wearing-Off”	Safety and efficacy as an adjunct to levodopa (60 mg oral daily)	II	Completed	
		NCT01283594^*^ (2011)	Motor fluctuations on levodopa	Safety and efficacy as an adjunct to levodopa(60, 120, 180, 240 mg twice/day)	II/III	Completed	Tozadenant (120 or 180 mg) was generally well tolerated and was effective at reducing off-time.
**BIIB014**	Oxford BioMedica	NCT00627588^*^ (2008)	Early Parkinson's disease	Safety, efficacy and dose evaluation	I/II	Completed	
**Caffeine**	McGill University Health Center	NCT01738178^*^ (2012)	Not specified	Motor effects of caffeine persist (or even magnify) helps reduce dose of other PD meds and/or prevents their side effects (200 mg daily)	III	Completed	-
	Ron Postuma	NCT01190735^*^ (2010)	Not specified	Optimal caffeine dose with maximal motor benefit and the least amount of undesirable adverse effects (100–200 mg twice/day)	II	Completed	
		NCT00459420^*^ (2007)	Not specified	Effect on sleepiness and motor symptoms (100–200 mg daily)	II/III	Completed	No significant benefit on excessive daytime sleepiness

* ClinicalTrials.gov

** *EU Clinical Trials Register*.

## Association of A_2A_AR antagonism and non-pharmacological approaches

Non-pharmacological approaches are strategies to combine, reinforce and complement the pharmacological options for the management and prevention of PD (Figure 1). Dance, treadmill and aquatic exercises feasibility to PD management have been evaluated in clinical trials with benefits to life quality, based in cognitive and motor features (Picelli et al., 2016; Carroll et al., 2017; Shanahan et al., 2017). Recently, it was demonstrated that treadmill exercises induce brain activation in PD (Maidan et al., 2017). These benefits have been reproduced in animal models of PD suggesting that physical exercise prevents the development of L-DOPA-induced dyskinesia and its association with hyperphosphorylation of DARPP-32, c-Fos expression and increased brain-derived neurotrophic factor (BDNF) levels (Gyárfás et al., 2010; Aguiar et al., 2013; Shin et al., 2017). Studies with wheel running rats revealed that A_1_AR and A_2A_AR expression is reduced in the striatum, reinforcing the idea that physical exercise is able to promote neuroplasticity and neuroprotection to brain regions related to motor control, probably through the reduction of antagonistic adenosine effects over dopamine signaling (Clark et al., 2014).

Deep Brain Stimulation (DBS) was approved by the FDA in 2002 as therapy for advanced PD (Suarez-Cedeno et al., 2017). From studies with animals, DBS appeared to have a neuroprotective effect against loss of dopaminergic neurons induced by classical dopaminergic neurotoxins (Maesawa et al., 2004). The use of A_2A_AR antagonism as an adjuvant of DBS in rodents suggests the potential to enhance the response in the treatment of parkinsonian symptoms, such as tremor (Collins-Praino et al., 2013). While clinical studies using transcranial direct current stimulation (tDCS) in PD suggest possible locomotor benefits, the biological mechanism is still under investigation (Benninger et al., 2011). In rodents, tDCS on the cerebral cortex promotes cognitive effects involving A_1_AR, although the adenosinergic participation in tDCS responses of PD has not been evaluated (Márquez-Ruiz et al., 2012). Electroconvulsive therapy (ECT) has been proposed to be efficient for both motor and non-motor symptoms in PD with psychological problems (Nishioka et al., 2014; Calderón-Fajardo et al., 2015). The proposed mechanism for ECT includes the enhancement of dopaminergic transmission in the striatum and an increase in the levels of levodopa by disrupting the blood–brain barrier (Kennedy et al., 2003). The purinergic system appears to be influenced by ECT, since the action, metabolism and release of nucleotide and nucleoside are altered under ECT, but no correlation with PD was identified until now (Gleiter et al., 1989; Busnello et al., 2008; Sadek et al., 2011). A combination of drugs and non-pharmacological therapies could warrant new investigations into the preclinical and clinical studies, with hope for the amelioration and affects in PD prevention, management and treatment.

## Conclusions

This review highlights the need to intensify research into adenosine signaling in the development of PD therapies. The interaction between adenosine and dopamine signaling has been extensively studied and contributed to knowledge of the role of non-dopamingergic neurotransmitters in the PD. As cholinergic, glutamatergic, GABAergic, canabinergic and serotoninergic systems appear together with adenosinergic system in the myriad of pathways involved in the PD, appearing together with the possibility of improved results from dual or multi-targeted anti-parkisonism approaches opened a new area of drug development. In addition, the association of pharmacological and non-pharmacological approaches brings new perspectives for a more effective treatment of PD and improved of quality of life for PD patients.

## Author contributions

LN, RdS, and CB equally contributed to the definition of the scope and to the writing of the manuscript.

### Conflict of interest statement

The authors declare that the research was conducted in the absence of any commercial or financial relationships that could be construed as a potential conflict of interest.

## Abbreviations

A_1_AR: A_1_ adenosine receptor
A_2A_AR: A_2A_ adenosine receptor
A_2B_AR: A_2B_ adenosine receptor
A_3_AR: A_3_ adenosine receptor
BDNF: brain-derived neurotrophic factor
DARPP-32: Dopamine- and cAMP-regulated phosphoprotein, Mr 32 kDa
D_1_DR: D_1_ dopamine receptor
D_2_DR: D_2_ dopamine receptor
PD: Parkinson's disease
6-OHDA: 6-hydroxydopamine
MPTP: 1-methyl-4-phenyl-1,2,3,6-tetrahydropyridine.
